# Arms Lift in a Case of Pseudoxanthoma Elasticum

**DOI:** 10.1155/2013/870605

**Published:** 2013-01-09

**Authors:** P. Panettiere, L. Marchetti, D. Accorsi

**Affiliations:** ^1^Dipartimento di Discipline Chirurgiche, Rianimatorie e dei Trapianti, Policlinico S.Orsola-Malpighi, Università degli Studi di Bologna, Via Massarenti 9, 40128 Bologna, Italy; ^2^Insegnamento di Chirurgia Plastica, Università degli Studi di Bologna, Via Massarenti 9, 40127 Bologna, Italy; ^3^Servizio di Chirurgia, Villa Chiara Hospital Via Porrettana 170, Casalecchio di Reno, 40033 Bologna, Italy

## Abstract

Pseudoxanthoma elasticum (PXE) is a rare hereditary disorder of elastin fibers, characterized by yellowish coalescent papules in flexural surfaces with abnormally lax and corrugated skin. It can be associated to systemic manifestations mostly regarding eyes and vessels. Aesthetic surgery of cutaneous hyperlaxity was described in the international literature only in few cases, mostly as neck lift. A 40-year-old woman presented with cutaneous signs of PXE, demanding brachioplasty. Results after a nine-month followup are quite satisfying, and no signs of local recurrence or scar alterations are present.

## 1. Introduction

Pseudoxanthoma elasticum (PXE) is a rare and heterogeneous hereditary disorder, characterized by frail and fragmented elastin fibers, with calcium deposits. The eyes, the arteries, and the skin are the main sites of clinical manifestations. Essentially two phenotypic varieties have been recognized: the first one with a prevalence of cutaneous lesions and minimal vascular and ophthalmic affections and the second one with relevant ocular and arterial lesions. The skin in the flexural surfaces is abnormally loose and creased, with yellowish papules, with strongly antiaesthetic features. Plastic surgery should therefore be an obvious therapeutic option for these patients. However, few cases of surgical treatment can be found in the literature, all substantially with good results and mostly reporting treatment of neck hyperlaxity.

## 2. Case Report

**Figure 1:**
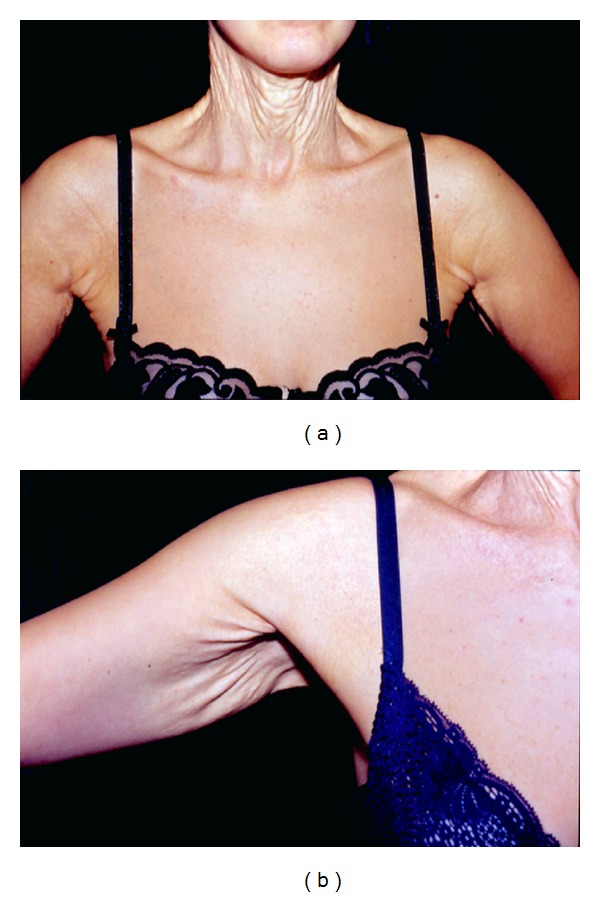
(a) Before surgery. Neck and armpit skin relaxation is evident. (b) Before surgery. Armpit detail.

**Figure 2:**
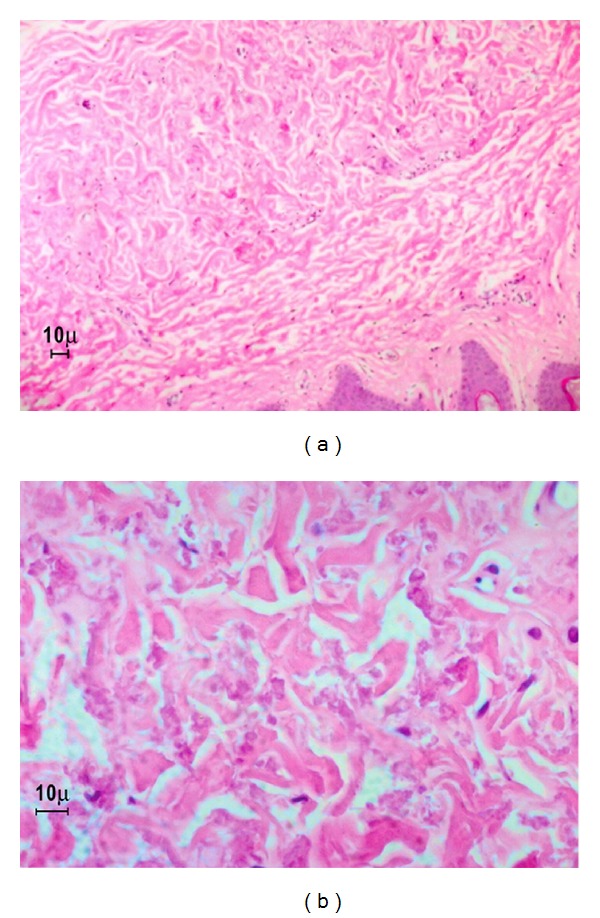
(a, b) Optical microscope (haematoxylin-eosin staining). Elastin fibers are distorted and altered with calcium deposits.

A 40-year-old patient came to our observation with typical skin features of PXE. No genetic disease was known in her family. When she was eight years old, she noticed the progressive appearance of yellowish papules on the skin of her neck, arms, and thighs. Subsequently, abnormal skin laxity became evident in the same areas, with severe aesthetic consequences (Figures 1(a) and 1(b)). PXE diagnosis was assessed by means of cutaneous biopsy, demonstrating remarkable fragmentation of dermal elastin fibers, with important calcium deposits (Figures 2(a) and 2(b)). Thorough ophthalmic and cardiologic examinations (including heart USG and retinal fluoroangiography) showed no associated disease. Color-Doppler of peripheral arteries demonstrated normal speed and pulse shape. 

**Figure 3:**
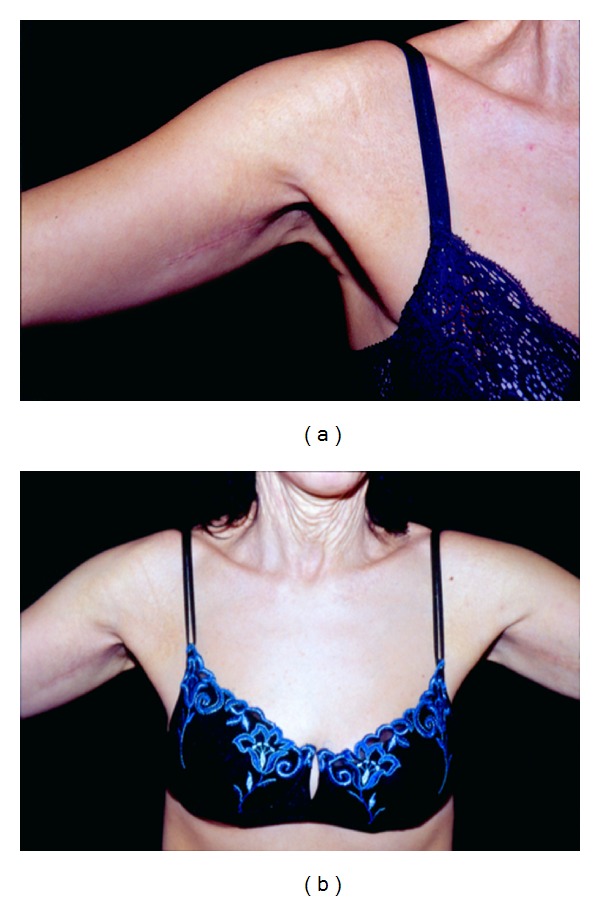
(a) One month after surgery. Recovery is still incomplete with reddish surrounding skin. (b) Six months after surgery. Good clinical recovery, with a thin linear scar.

Even if neck laxity was the most evident lesion, the patient did not feel much social discomfort about this site, as “*all her friends knew her problem*.” On the contrary, she felt much embarrassment about her armpits and thighs. However, her concern about scars and the need for long resting after thighs lift made her opt for arms hyperlaxity treatment only. So, she underwent arms lifting under local anesthesia with the excision of two skin sectors of approximately 15 × 6 cm. The main technical concern was the extreme weakness and thinness of the dermal layer that made suture extremely tricky. In particular, many more stitches (3/0 and 4/0 polyglyconates) than usual were demanded, also due to the considerable skin rigidity. Tightening up the skin to fit the new tension state was also particularly difficult. Some loose 3/0 polyglyconate stitches were placed about 5 mm far from each wound margin to approximate and raise the margins, in order to reduce the tension applied to the superficial stitches and prevent scar widening. No bleeding problems occurred, and wound healing was substantially normal, even if slightly delayed (Figure 3(a)). Nine months after surgery, fully satisfactory functional and aesthetic results were achieved (Figure 3(b)), and the patient is now considering neck and thighs lifting. 

## 3. Discussion

Pseudoxanthoma elasticum (PXE) is a heterogeneous, essentially genetic (autosomal dominant and autosomal recessive varieties) elastin disorder. Acquired cases due to penicillamine administration were also described [1]. Genetic varieties have been associated to 43 distinct mutations of gene ABCC6 (ATP-binding cassettes subfamily C member 6) [2]. All the varieties are characterized by elastin abiotrophy with highly fragmented and aggregate fibers, on which calcium crystals deposit. Even if almost all tissues are potentially involved, the most frequently affected organs are eyes (angioid streaks, retinal hemorrhages, and myopia), cardiovascular system (diminished peripheral pulse, hypertension, congestive cardiac failure, coronary artery disease, mitral valve prolapse, arteriosclerosis [3], subarachnoid hemorrhages), and skin (hyperlaxity of flexural surfaces with coalescent yellowish papules). Less frequently, gynecologic (uterus and bladder prolapse) and gastrointestinal problems (digestive hemorrhages) have been reported.

Two distinct clinical varieties have been described, one with prevailing cutaneous lesions and moderate systemic manifestations (autosomal recessive Pope's type I) and the other with severe systemic lesions and minimal cutaneous signs (autosomal dominant and Afrikaaner varieties).

Cutaneous lesions are nearly invariably limited to the neck, the arms, and the thighs, even if abnormal breast and abdomen laxity have also been described [3]. Loose skin can be treated with plastic surgery, but few works can be found in the literature about surgical options for this rare disease, the majority of which are regarding neck hyperlaxity [3–8]. Brachioplasty was described in only two cases [3]. Aesthetic results reported by the authors are generally satisfactory, and only in few patients, wound widening have been described. 

The case presented here is emblematic because clinical manifestations were mainly confined to the skin of neck, arms, and thighs, though they appeared earlier than usual. The patient opted for arms treatment only, and a classic technique brachioplasty was therefore performed. The main technical concern was dermal weakness and inelasticity, as wound margins approximation by sutures could be extremely difficult and unstable. In the wider series published, no particular surgical trouble was described [3]. In the present case, on the contrary, we observed serious practical difficulties conflicting with such assertions. Suturing problems were resolved by applying more stitches than usual and by placing some loose ones quite distant from the margins in order to reduce tension. Elastin defect and calcification in the walls of small arteries could be potentially responsible for poor postretraction haemostasis, and excessive bleeding could be expected. On the contrary, no such problems occurred, as described also in other works about surgery in PXE patients [3].

Wound repair was substantially normal, even if slightly slower than usual. Calcified nodules expulsion described by some authors did not occur [3].

Nine months after surgery, the results are fully satisfactory and encourage to assert that plastic surgery should be considered as a valid option in the treatment of the cutaneous lesions of PXE. 

## References

[1] Narron GH, Zec N, Neves RI, Manders EK, Sexton FM (1992). Penicillamine-induced pseudoxanthoma elasticum-like skin changes requiring rhytidectomy. *Annals of Plastic Surgery*.

[2] Ohtani T, Furukawa F (2002). Pseudoxanthoma elasticum. *The Journal of Dermatology*.

[3] Vijoen DL, Bloch C, Beighton P (1990). Plastic surgery in pseudoxanthoma elasticum: experience in nine patients. *Plastic and Reconstructive Surgery*.

[4] Chen TH, Wei FC (1998). Pseudoxanthoma elasticum. Case report. *Scandinavian Journal of Plastic and Reconstructive Surgery and Hand Surgery*.

[5] Crickelair GF (1953). Pseudoxanthoma elasticum treated surgically. *Plastic and Reconstructive Surgery*.

[6] Kaplan EN, Henjyoji EY (1976). Pseudoxanthoma elasticum: a dermal elastosis with surgical implications. *Plastic and Reconstructive Surgery*.

[7] Ng ABY, O’Sullivan ST, Sharpe DT (1999). Plastic surgery and pseudoxanthoma elasticum. *British Journal of Plastic Surgery*.

[8] Pickrel KL, Kelley JW, Marzoni FA (1948). The plastic surgical treatment of pseudoxanthoma elasticum. *Plastic and Reconstructive Surgery*.

